# Supplementary material for: Getting psychiatry on the move—Implementation and evaluation of Braining, a structured physical exercise intervention in outpatient psychiatry: A convergent-parallel mixed methods study
Source: PLoS One. 2026 May 21;21(5):e0348234. doi: 10.1371/journal.pone.0348234 (PMC13193532; doi:10.1371/journal.pone.0348234)
Supplement: S1 Appendix — (DOCX) [file pone.0348234.s001.docx]

**SUPPLEMENTAL FILE 1: Quantitative survey including the Acceptability of Intervention Measure (AIM), Intervention Appropriateness Measure (IAM), and Feasibility of Intervention Measure (FIM)**

**GENERAL INSTRUCTIONS:** These measures could be used independently or together. The IAM items could be modified to specify a referent organization, situation, or population (e.g., my clients). Please check and report the psychometric properties with each use or modification.

**Acceptability of Intervention Measure (AIM)**

|  | **Completely disagree** | **Disagree** | **Neither agree nor disagree** | **Agree** | **Completely agree** |
| --- | --- | --- | --- | --- | --- |
| **1. (INSERT INTERVENTION) meets my approval.** | □ | □ | □ | □ | □ |
| **2. (INSERT INTERVENTION) is appealing to me.** | □ | □ | □ | □ | □ |
| **3. I like (INSERT INTERVENTION).** | □ | □ | □ | □ | □ |
| **4. I welcome (INSERT INTERVENTION).** | □ | □ | □ | □ | □ |

**Intervention Appropriateness Measure (IAM)**

|  | **Completely disagree** | **Disagree** | **Neither agree nor disagree** | **Agree** | **Completely agree** |
| --- | --- | --- | --- | --- | --- |
| **1. (INSERT INTERVENTION) seems fitting.** | □ | □ | □ | □ | □ |
| **2. (INSERT INTERVENTION) seems suitable.** | □ | □ | □ | □ | □ |
| **3. (INSERT INTERVENTION) seems applicable.** | □ | □ | □ | □ | □ |
| **4. (INSERT INTERVENTION) seems like a good match.** | □ | □ | □ | □ | □ |

**Feasibility of Intervention Measure (FIM)**

|  | **Completely disagree** | **Disagree** | **Neither agree nor disagree** | **Agree** | **Completely agree** |
| --- | --- | --- | --- | --- | --- |
| **1. (INSERT INTERVENTION) seems implementable.** | □ | □ | □ | □ | □ |
| **2. (INSERT INTERVENTION) seems possible.** | □ | □ | □ | □ | □ |
| **3. (INSERT INTERVENTION) seems doable.** | □ | □ | □ | □ | □ |
| **4. (INSERT INTERVENTION) seems easy to use.** | □ | □ | □ | □ | □ |

**Pragmatic Qualities:**

- Readability tested by substituting “This EBP” for “Insert Intervention.” Flesch reading ease score (and grade level) is 95.15 (5^th^ grade) for AIM, 99.60 (5^th^ grade) for IAM, and 94.17 (5^th^ grade) for FIM.
- No specialized training is needed to administer, score, or interpret the measures.
- Cut-off scores for interpretation not yet available; however, higher scores indicate greater acceptability, appropriateness, or feasibility.
- Norms not yet available.
- Scales can be created for each measure by averaging responses. Scale values range from 1 to 5. No items need to be reverse coded. Good measurement practice: assess structural validity to confirm the unidimensionality of each measure and calculate alpha coefficient to ascertain reliability.
- There is no cost to use these measures.
- Time to complete: less than 5 minutes per measure.
