# Supplementary material for: Getting psychiatry on the move—Implementation and evaluation of Braining, a structured physical exercise intervention in outpatient psychiatry: A convergent-parallel mixed methods study
Source: PLoS One. 2026 May 21;21(5):e0348234. doi: 10.1371/journal.pone.0348234 (PMC13193532; doi:10.1371/journal.pone.0348234)
Supplement: S2 Appendix — (DOCX) [file pone.0348234.s002.docx]

**SUPPLEMENTAL FILE 2: Focus group discussion guide 1 (translated from Swedish) used in focus groups 1 and 2, 4 months after intervention start point**

**Introduction to Braining**

1. To start off, tell me about your experience working with Braining.

In the introduction to Braining, you were invited to participate in an introductory presentation about Braining followed by a full-day education meeting (the so-called “Braining Day), that included a one-hour joint information session with all staff, a three-hour workshop with the Braining team, and an afternoon session solely for Braining instructors and hosts.

1. How did you experience the introduction you received?
2. What was your attitude towards Braining before you started working with the intervention?
   - What was your general attitude towards physical activity as a form of treatment?
3. How did you perceive other staff’s attitudes towards Braining before you started working with the intervention?
   - How did you perceive your colleagues’ general attitude towards physical activity as a form of treatment?

**Working with Braining on a Day-to-Day Basis**

1. How have you introduced Braining into your daily work?
2. What were the factors that made it possible to start working with Braining? What were the factors that made it hard to start working with Braining?
   - Prompts: e.g., time, resources, support from manager, support from research team, etc.
3. What advantages do you see with Braining? (for staff, for patients)
4. What challenges do you see with Braining? (for staff, for patients)
   - Possible prompts for Q7 & Q8: collaboration, time required, synergistic effects, competing priorities, work prioritisation…
5. Do you feel that working with Braining has led to any changes in your unit? If so, in what way?
6. How do you monitor that you are working according to Braining? (e.g. track progress, follow-up on key activities, etc.)
7. Earlier, you were asked about some of the challenges you have encountered with Braining. How have you managed these challenges?
   - Prompts: e.g., solutions, go-to person, staff meetings, Braining team
8. Earlier, you were about how you monitor that you are working according to Braining. How do you provide feedback about Braining’s progress to other staff and managers?

**Looking ahead**

1. How would you describe the general attitude towards Braining among staff today, after you have worked with Braining for a while?
2. If you could change anything about Braining, fully or partly, what would you do to make Braining work more effectively?
3. Do you believe that Braining could be used in psychiatry in its present form and/or following the adaptations you suggested?
   - Prompts: Or are there other ways once can support physical activity?
4. Now that you have talked about and reflected upon Braining’s introduction, how you work with Braining on a day-to-day basis, and its future potential, is there anything else you would like to add?
