# Supplementary material for: Getting psychiatry on the move—Implementation and evaluation of Braining, a structured physical exercise intervention in outpatient psychiatry: A convergent-parallel mixed methods study
Source: PLoS One. 2026 May 21;21(5):e0348234. doi: 10.1371/journal.pone.0348234 (PMC13193532; doi:10.1371/journal.pone.0348234)
Supplement: S3 Appendix — (DOCX) [file pone.0348234.s003.docx]

**SUPPLEMENTAL FILE 3: Focus group discussion guide 2 (translated from Swedish) used in focus group 3, 12 months after intervention start point**

1. What is your role in the Braining team?
2. Some of you met with me in a prior focus group. How have things gone since then?
3. How do you perceive the level of engagement and work within the Braining team?
4. Do you feel that Braining is currently a natural part of your work?
   - What has helped you to achieve that?
   - What have been some of the challenges you have encountered to achieve that?
5. Do you feel that other staff are involved in informing patients about Braining and recruiting them to Braining?
6. How have you experienced working with core components 1–7? (address each core component individually)
7. What adaptations have been made to the core components?
   - How have you adapted the core components?
   - Why and/or for whom have you adapted the core components? (e.g., staff/patients)
   - When was the adaptation made?
   - What impact has each adaptation had? Have the adaptation had any unintended consequences/effects?
8. In our earlier focus group discussions, difficulties recruiting patients (especially younger patients) were raised. How has that worked out since then?
   - a. What adaptations have you made related to this? (e.g., adaptations for staff?)
9. How do tailor or adapt the Braining sessions for your patients? Is it possible?
10. How do you assess whether a patient is suitable to participate in Braining? Are there patients who are unsuitable for Braining?
11. Have other tasks been impacted as a result of your work with Braining?
12. In our earlier focus group discussions, the risk of Braining’s implementation being dependent on champions (key members of staff) was raised. How have things worked out since then? How do you currently view this issue?
13. How do you ensure that you will continue to deliver Braining in your unit, despite high staff turnover? (both within the Braining team and among other staff)
14. Has your involvement in the research study impacted engagement towards Braining? If the research study influenced Braining’s implementation, how have you experienced running Braining more independently?
15. How has Braining impacted your unit?
16. Is there anything else you would like to add?
