# Supplementary material for: Getting psychiatry on the move—Implementation and evaluation of Braining, a structured physical exercise intervention in outpatient psychiatry: A convergent-parallel mixed methods study
Source: PLoS One. 2026 May 21;21(5):e0348234. doi: 10.1371/journal.pone.0348234 (PMC13193532; doi:10.1371/journal.pone.0348234)
Supplement: S4 Appendix — (DOCX) [file pone.0348234.s004.docx]

**S4 Appendix. List of abbreviations**

**AIM** Acceptability of Intervention Measure

**FIM** Feasibility of Intervention Measure

**FGD** Focus group discussion

**HCW** Healthcare worker

**IAM** Intervention Appropriateness Measure

**PE** Physical exercise

**SUD** Substance use disorders
