# Supplementary material for: Getting psychiatry on the move—Implementation and evaluation of Braining, a structured physical exercise intervention in outpatient psychiatry: A convergent-parallel mixed methods study
Source: PLoS One. 2026 May 21;21(5):e0348234. doi: 10.1371/journal.pone.0348234 (PMC13193532; doi:10.1371/journal.pone.0348234)
Supplement: Attachment — Submitted filename: Response letter to reviewers PLOS ONE.docx [file pone.0348234.s006.docx]

# Response letter

We would like to thank the reviewers for their thoughtful and constructive comments, and the editor for the opportunity to revise our manuscript. We have carefully considered each comment and made every effort to address them thoroughly and appropriately. Below, we provide a point-by-point response to all reviewer comments. A revised version of the manuscript with changes marked in yellow is enclosed for ease of review.

**Reviewer #1:**

Thank you for the opportunity to review this manuscript. This is a well-written and conceptually strong mixed-methods implementation study evaluating the acceptability, appropriateness, and feasibility of the “Braining” physical exercise intervention within Swedish outpatient psychiatry. The manuscript is clear, methodologically transparent, and presents a coherent integration of qualitative and quantitative findings. It makes an important contribution to implementation research in mental health—an area where evidence-based physical exercise interventions remain underutilized.

That said, several areas could be strengthened to enhance the manuscript

**Response to reviewer 1:** We thank the reviewer for their positive and encouraging assessment of our manuscript. We have carefully considered the suggestions for improvement outlined below and have revised the manuscript accordingly.

1. **Statistical analysis of normality**
   The authors note that both mean and median values were calculated due to the small sample size and potential skewness. This issue could be addressed more rigorously by performing a formal test of normality (e.g., Kolmogorov–Smirnov or Shapiro–Wilk test). Reporting this would provide stronger justification for the use of descriptive statistics and interpretation of central tendency.

**Response**: Thank you for your valuable comment regarding the statistical approach and data distribution. We have now performed a Shapiro–Wilk test of normality, which indicated that data for all outcome measures deviated significantly from a normal distribution (W = 0.734-0.860, p =.001 - .049). Consequently, we chose not to report means and standard deviations in the descriptive statistics. Instead, medians and interquartile ranges (IQRs) are presented. This information has been added to ease interpretation of the descriptive statistics.

See revisions in manuscript:

Line 151: A Shapiro–Wilk test of normality was performed and indicated that data for all three outcome measures deviated significantly from a normal distribution (W = 0.734-0.860, p =.001 - .049). Therefore, descriptive statistics for each measure were presented, including median, interquartile range (IQR), and response rates.

Line 209: Acceptability

Line 273: Appropriateness

Line 343: Feasibility

2. **Discussion**

   **a.** The discussion would benefit from substantial revision to reduce redundancy and deepen analytical interpretation. Several themes—such as initial staff skepticism followed by enthusiasm, positive patient responsiveness, and difficulty engaging younger patients—are repeated multiple times across sections. Condensing these will improve readability and allow more space to discuss the implications of findings within implementation science frameworks.

**Response:** We thank the reviewer for this constructive and helpful comment. We have substantially revised the Discussion to reduce redundancy and strengthen analytical interpretation. Repeated themes, such as initial staff scepticism followed by increased engagement, positive patient responsiveness, and challenges in engaging younger patients, have been consolidated across sections and domains to improve readability and coherence. This restructuring has allowed us to focus more clearly on the implications of the findings and to deepen the analysis by explicitly situating the results within an implementation science framework. In particular, we have applied CFIR constructs to interpret the observed implementation outcomes.

In line with this strengthened analytical focus, we have also revised the study aim to clarify that the study sought not only to explore but also to explain healthcare workers’ perceptions of acceptability, appropriateness, and feasibility. This change reflects the more explicit use of implementation theory to interpret and contextualise the findings.

See full discussion from line 463.

Se revised aim on line 30 and 99:

This study sought to explore and explain healthcare workers’ acceptability, appropriateness, and feasibility of Braining in an outpatient psychiatric setting to inform future implementation efforts.

**Response to comments b-e:** We thank the reviewer for these related comments. We have carefully reviewed the manuscript and revised the wording throughout to clarify that the reported findings reflect participants’ observed experiences and perceptions, rather than inferred causal relationships. Statements that could previously be interpreted as implying causality have been rephrased to explicitly describe experiential or observational accounts. These clarifications have been applied consistently across all examples raised in comments b–e and are indicated in the revised manuscript.

**b.** The authors identify motivational work as a key component of Braining’s success. While plausible, the current study design does not allow for conclusions about causal or mechanistic effects. A process evaluation or implementation fidelity assessment would be required to identify which components of Braining are essential to its success. The discussion should be revised to reflect this limitation.

**Response:** We thank the reviewer for this important clarification. We agree that the current study design does not allow conclusions regarding causal or mechanistic effects of specific Braining components. We have therefore revised the Discussion to explicitly frame motivational work as perceived by staff rather than as a demonstrated driver of outcomes. In addition, we have added a clear methodological limitation, noting that a process evaluation or implementation fidelity assessment would be required to identify which components of Braining are essential for patient engagement and retention. These revisions are reflected in the updated Discussion section *Individuals domain*:

Line 505:

At the 12-month follow-up, HCWs described Braining as integrated into routine practice at participating clinics, indicating its relative priority, mission alignment, and compatibility were embedded in everyday work. This reflects local routinisation rather than formal adoption at the organisational or system level, which was not evaluated. Participants identified factors for continued delivery: a stable multidisciplinary Braining team, strong managerial endorsement, and the incorporation of tasks into existing workflows. These align with recommendations for sustaining lifestyle interventions in mental health care through organisational prioritisation, leadership support, and service integration (39). However, adoption and longer-term sustainability change remain to be examined.

**c.** The statement “HCWs observed notable improvements in patients’ quality of life and lifestyle behaviours” should be clarified. It is not clear how these improvements were measured or defined. “Quality of life” typically requires a validated, operationalized measure (e.g., WHOQOL-BREF, SF-36). If this was anecdotal or based on qualitative impressions, it should be presented as such.

**Response:** We thank the reviewer for this important comment. We agree that the original wording could be interpreted as implying intervention effects beyond the scope of the study. We have therefore revised both the Results and Discussion sections to clarify that these findings reflect HCWs’ observations and interpretations of patient-reported experiences, rather than demonstrated effects of Braining. In the Discussion, we have further framed these observations within the construct of appropriateness, emphasizing perceived relevance and fit for the patient group rather than causal outcomes. All related statements have been revised accordingly.

Line 41 Results in Abstract: HCWs perceived improvements in patients’ quality of life, benefits of joint staff–patient training sessions and social connectedness.

Line 316 Results:

*Perceived changes in patients’ quality of life and lifestyle behaviours*

HCWs described perceived improvements in patients’ quality of life following participation in Braining. Staff shared accounts of patients reporting substantial personal benefits, which were described as profound in some cases; one patient stated that they would not have survived without Braining, while another reported that participation supported continued abstinence from substances and enabled admission to further treatment. According to HCWs, patients also described Braining as supportive of broader lifestyle changes. These observations were based on staff reflections and patient narratives shared during the intervention period.

Line 566 Discussion:

HCWs perceived benefits in patients’ quality of life and lifestyle following participation in Braining. They reported that some patients credited the programme with profound personal and social benefits, with the social value particularly beneficial for individuals with social anxiety, as it enabled them to expand their networks and develop a sense of belonging. These observations are consistent with systematic reviews showing that exercise interventions can improve quality of life, mood, and social functioning in people with SUD, particularly in group-based formats that foster social support (8,12,38–40). Importantly, although PE has been associated with reductions in consumption, cravings and withdrawal symptoms (37,49,50), evidence regarding effects on substance use outcomes remains mixed. It may be contingent on how the intervention is designed and implemented. Consequently, while Braining appears to offer meaningful personal and social benefits, conclusions regarding its causal impact on patients’ substance use and broader lifestyle outcomes cannot yet be drawn; further investigation is needed.

**d.** The claim that “structured weekly meetings played a crucial role in sustaining Braining’s implementation” is interesting but cannot be substantiated by this study design. The authors may report that participants perceived these meetings as important, but attributing causal impact requires a process or outcome evaluation. Please revise this phrasing accordingly.

**Response:** We thank the reviewer for this important clarification. We agree that the study design does not allow conclusions regarding causal effects of structured weekly meetings. We have therefore revised the wording to explicitly reflect participants’ perceptions of these meetings as important for implementation, rather than attributing a causal role. The revised phrasing is now framed in experiential terms and no longer implies mechanistic impact:

Line 551: A core Braining component is the multidisciplinary implementation team, which convenes regular meetings with a predefined agenda to ensure coordinated delivery. Participants emphasised this team's pivotal role in both initiating and sustaining the innovation through teaming, where shared responsibility and stable processes enable consistent implementation.

**e.** The study was designed to evaluate acceptability, appropriateness, and feasibility of Braining, not to determine which components of Braining drive effectiveness. The discussion should explicitly acknowledge this scope and avoid making inferences about which aspects (e.g., teamwork, motivation) are “key” to success without supporting evidence.

**Response:** We thank the reviewer for this important clarification. We have revised the Discussion to more explicitly acknowledge the study’s scope, emphasising that the evaluation focused on acceptability, appropriateness, and feasibility rather than on identifying causal or mechanistic drivers of effectiveness. Statements that could be interpreted as implying that specific components of Braining were “key” to success have been rephrased to reflect perceived relevance and associations rather than demonstrated effects. We have also clarified that determining which components contribute most strongly to outcomes would require dedicated process evaluations or effectiveness studies.

See full discussion and Line 610:

While the findings elucidate implementation factors, they cannot address causal or mechanistic effects; a process evaluation or fidelity assessment would be required. Results thus reflect perceived relevance rather than demonstrated effectiveness of Braining.

**f.** The discussion would benefit from clarification on whether Braining has now been adopted as part of standard care in the participating clinics. What mechanisms have been put in place to ensure this?

**Response:** We thank the reviewer for this important clarification. We have revised the Discussion to explicitly state that healthcare workers described Braining as integrated into routine practice at the participating clinics at the time of follow-up, while clarifying that formal adoption as standard care and long-term sustainability were not evaluated in this study. We also describe the locally perceived mechanisms supporting continued delivery, including a stable multidisciplinary team, managerial endorsement, and integration of Braining-related tasks into existing clinical workflows.

Line 505:

At the 12-month follow-up, HCWs described Braining as integrated into routine practice at participating clinics, indicating its relative priority, mission alignment, and compatibility were embedded in everyday work. This reflects local routinisation rather than formal adoption at the organisational or system level, which was not evaluated. Participants identified factors for continued delivery: a stable multidisciplinary Braining team, strong managerial endorsement, and the incorporation of tasks into existing workflows. These align with recommendations for sustaining lifestyle interventions in mental health care through organisational prioritisation, leadership support, and service integration (39). However, adoption and longer-term sustainability change remain to be examined.

**Reviewer #2:**

Moving Psychiatry Forward – Implementation and evaluation of Braining, a structured
physical exercise intervention in outpatient psychiatry: a convergent-parallel mixed
methods study
This research examined an important issue that will add valuable insights to the body of knowledge. The methodology is sound, and the paper is well written with its strengths and limitations well captured. I have few observations which are highlighted:

**Response to reviewer 2:** We thank the reviewer for their positive and thoughtful evaluation of our manuscript. We appreciate the recognition of the study’s relevance, methodological rigor, and balanced presentation of strengths and limitations. Below, we address the reviewer’s observations point by point and describe how the manuscript has been revised accordingly.

1. Title: While the paper provides excellent insight into implementation of structured physical exercise in outpatient psychiatry clinics, adding the caption of “moving psychiatry forward” is framed too broadly than the scope of the study. Consider revising the title to accurately reflect the study’s boundaries.

**Response:** We thank the reviewer for this helpful comment. We agree that the original title may have framed the study's scope too broadly. To better reflect the study’s boundaries while retaining a descriptive and engaging phrasing, we have revised the title to:

Line 1: Getting psychiatry on the move – Implementation and evaluation of Braining, a structured physical exercise intervention in outpatient psychiatry: a convergent-parallel mixed methods study.

2. Methodology: It will be helpful to include the psychometric properties of the validated instruments used {AIM – acceptability of Intervention Measure (AIM); IAM – Intervention Appropriateness Measure (IAM); FIM – Feasibility of Intervention Measure (FIM)}.

**Response**: We thank the reviewer for this suggestion. We have now added descriptions of the psychometric properties of AIM, IAM, and FIM, including internal consistency, factor structure, and construct validity, with references to the original validation study by Weiner et al. (2017).

Line 129:

Quantitative data were collected using the Swedish version of the Acceptability of Intervention Measure (AIM), Intervention Appropriateness Measure (IAM), and Feasibility of Intervention Measure (FIM), validated implementation outcome measures developed for use in implementation research. Each scale consists of four items rated on a five-point Likert scale, yielding total scores ranging from 4 to 20. In the original psychometric evaluation, all three measures demonstrated good to excellent internal consistency (Cronbach’s α = 0.85–0.91), a unidimensional factor structure, and evidence of construct validity, supporting their use as distinct but related early-stage implementation outcomes (28).

3. Results
   This statement in line 228 to 231 appears complex and difficult to understand. The statement suggests that those outside the Braining Team assume the intervention will target staff rather than patients and on the contrary patient’s interest in Braining was perceived as high….

**Response:** We thank the reviewer for highlighting this issue. We agree that the original statement was complex and have revised the wording to improve clarity and readability. The revised text now more clearly distinguishes between staff expectations regarding Braining’s target group and the observed high level of patient interest.

Line 224:

The participants were surprised that some colleagues outside the Braining team showed limited interest in the intervention. They had initially expected Braining to appeal more to staff than to patients; however, patient interest was described as generally high, with few exceptions. The Braining team reflected that a more engaging introduction of Braining to all staff might have increased staff interest and engagement. They also identified additional factors that may have influenced staff acceptability of Braining, which are described below.

4. Discussion: Line 584 to 681- This statement here seems to be outside the scope of what this study addressed, with no clue from the methodology or result pointing to this.

**Response:** We thank the reviewer for this important comment. We agree that the original wording could be interpreted as implying intervention effects beyond the study's scope. We have therefore revised the section to clarify that these findings reflect HCWs’ observations and interpretations of patient-reported experiences, rather than demonstrated effects of Braining. In the Discussion, we have further framed these observations within the construct of appropriateness, emphasising perceived relevance and fit for the patient group rather than causal outcomes.

Line 566:

HCWs perceived benefits in patients’ quality of life and lifestyle following participation in Braining. They reported that some patients credited the programme with profound personal and social benefits, with the social value particularly beneficial for individuals with social anxiety, as it enabled them to expand their networks and develop a sense of belonging. These observations are consistent with systematic reviews showing that exercise interventions can improve quality of life, mood, and social functioning in people with SUD, particularly in group-based formats that foster social support (8,12,38–40). Importantly, although PE has been associated with reductions in consumption, cravings and withdrawal symptoms (37,49,50), evidence regarding effects on substance use outcomes remains mixed. It may be contingent on how the intervention is designed and implemented. Consequently, while Braining appears to offer meaningful personal and social benefits, conclusions regarding its causal impact on patients’ substance use and broader lifestyle outcomes cannot yet be drawn; further investigation is needed.
