# Supplementary material for: Getting psychiatry on the move—Implementation and evaluation of Braining, a structured physical exercise intervention in outpatient psychiatry: A convergent-parallel mixed methods study
Source: PLoS One. 2026 May 21;21(5):e0348234. doi: 10.1371/journal.pone.0348234 (PMC13193532; doi:10.1371/journal.pone.0348234)
Supplement: Attachment — Submitted filename: Response letter PLOS ONE 260405.docx [file pone.0348234.s007.docx]

# Response letter

**Reviewer #1**

I thank the authors for their efforts and careful revision of the manuscript. The current draft is substantially improved compared with the previous version. However, several issues still need to be addressed.

**Response to reviewer 1:** We sincerely thank the reviewer for their careful evaluation of our revised manuscript and for their constructive and encouraging feedback. We appreciate the recognition that the manuscript has been substantially improved. We have carefully addressed all remaining comments and revised the manuscript accordingly to further enhance clarity, precision, and transparency. Our detailed responses are provided below, and the changes in the manuscript are marked in yellow.

1. Line 162: “Only Braining team members agreed to participate (n=8).” Please ensure that the reporting of the qualitative results and the Discussion consistently reflect this. Using the term “HCWs” throughout may be misleading regarding who contributed qualitative data. You note this in the Limitations, which is helpful; however, the wording in Results/Discussion should also be consistently aligned.

**Response**: We thank the reviewer for this important clarification. We have revised the manuscript throughout the **Results (line 197)** and **Discussion (line 467)** to consistently distinguish between healthcare workers (HCWs) in general and the specific focus group discussion participants (Braining team members, n=8). As a result, wording has been updated in multiple places and marked in yellow to ensure that the source of each finding is described accurately and consistently.

2. Lines 201–202: “Of the 29 HCWs working at the units, a total of 16 participated in this study. Specifically, 14 HCWs completed the surveys, of whom 8 also participated in the FGDs.” This description is unclear as written. Please clarify the total number of participants.

**Response:** We thank the reviewer for highlighting this lack of clarity. We have revised the text to explicitly report the total number of participants and the overlap between survey and qualitative data. The revised description now clarifies how many participants contributed to each component and how many participated in both, to ensure that the sample is transparent and easy to interpret.

Line 201-204: Of the 29 HCWs working at the units, 16 participated in the study: 14 completed the surveys, eight participated in the FGDs, and six contributed to both. Consequently, eight participants provided survey data only, two provided qualitative data only, and six contributed to both components.

3. Lines 551–553: The claim that check-ins benefit many but may feel intrusive and that motivational support should be balanced with patient autonomy is broadly consistent with the Results. However, consider replacing “intrusive” with wording closer to the data (e.g., “unnecessary”/“burdensome”), unless you have verbatim support for “intrusive,” to avoid any perception of over-interpretation.

**Response:** We agree and have revised the wording to better reflect the empirical data. The term “intrusive” has been replaced with “unnecessary” to avoid potential over-interpretation.

Line 552-554: Although regular check-ins were beneficial for many patients, others found them unnecessary, again underscoring the importance of assessing needs to balance motivational support with patient autonomy.

4. Lines 568–570: “These forums also enabled reflection and evaluation through progress reviews and barrier discussions, facilitating adaptation, a core mechanism for sustaining implementation in dynamic clinical settings.” The Results support that weekly meetings were structured, agenda-driven, and used for progress monitoring/coordination. However, the manuscript does not currently provide clear empirical examples of adaptations arising from these meetings, nor does it measure sustainability mechanisms. Please consider tightening this statement to what is directly supported and/or add concrete examples of adaptations from the FGDs. If you retain “adaptation” and “sustaining implementation,” frame this more explicitly as interpretive/hypothesis-generating and support it with appropriate implementation literature.

**Response:** We thank the reviewer for this important observation. We have revised this section to more closely reflect the empirical data and to avoid overinterpretation. The description of the meetings has been adjusted to align with the Results, emphasising their structured and coordinating role. Where reference to adaptation is retained, it is now more cautiously framed as an interpretive perspective (e.g., “appeared to” / “may have supported”), rather than a directly measured mechanism. We have also clarified the link to implementation literature regarding adaptation and sustaining implementation.

Lines 567–573: The structure of regular meetings appeared to support the team in assessing needs (patient-level and organisational challenges) and context (local workflows, resources, unit priorities), planning (coordinated recruitment, delivery, and barrier management), and tailoring strategies (procedure adjustments based on insights). These forums also enabled reflection and evaluation through progress reviews and discussions of barriers, which may have supported ongoing adaptation processes. Such processes are considered important for sustaining implementation in dynamic clinical settings (35).

5. Line 580: The statement that evidence regarding effects on substance use outcomes is “mixed” requires an explicit citation. If the sources already cited in the paragraph support this claim, attach them directly to this sentence; otherwise, please add the most appropriate citations.

**Response:** We thank the reviewer for this important comment. We have revised the sentence to more precisely reflect the evidence base and to ensure that the claim is directly supported by appropriate citations. Specifically, we have clarified that while exercise has been associated with improvements in craving and withdrawal symptoms, effects on substance use outcomes are heterogeneous, and we have aligned the cited references to directly correspond to these distinct aspects of the evidence.

Lines: 581-585: Importantly, although PE has been associated with improvements in craving and withdrawal symptoms (37,48), effects on alcohol and other SUD outcomes are heterogeneous across studies, with some trials and meta-analyses reporting limited or no impact on consumption or binge drinking (49,50).

6. Lines 590–593: “An additional and unanticipated outcome was enhanced collaboration across units… improved referral processes across services…” This interpretation is largely supported by the qualitative results. Two refinements are recommended: (1) qualify that these are participant-reported perceptions from Braining team members, not unit-wide staff, and (2) consider narrowing “across services” to “between the two units” unless you have evidence of broader service-level effects. In addition, because this is in the Discussion, it would be strengthened by grounding the observation in relevant organizational/leadership theory and discussing implications.

**Response:** We thank the reviewer for this helpful comment. We have revised this section to clarify that these findings reflect participant-reported perceptions from Braining team members, rather than unit-wide staff. We have also narrowed the wording from “across services” to “between the two units,” as suggested. In addition, we have strengthened the discussion by briefly grounding these observations in organisational and implementation perspectives, highlighting the potential role of collaboration and coordination in supporting implementation.

Lines 597-602: An additional and unanticipated outcome, as reported by participants, was enhanced collaboration between the two units. According to participants, Braining strengthened peer-to-peer relationships and improved referral processes, suggesting that innovation may confer organisational benefits beyond patient-level effects. Such improvements in collaboration and coordination are consistent with implementation literature emphasising the role of organisational context and interprofessional collaboration in successful implementation (35).

7. Lines 594–598: This passage is currently vague. It is unclear what “these findings” refer to, particularly given that the preceding paragraph focuses on cross-unit collaboration. If this section is intended to extend the discussion of perceived work-related benefits of Braining, please strengthen the transition and make explicit that these are perceived benefits reported by Braining team participants. In its current form, the text risks implying measurement of job satisfaction, stress, or workload that was not directly assessed. Also, please verify the citation; I believe the appropriate Quadruple Aim reference is: Bodenheimer T, Sinsky C. From triple to quadruple aim: care of the patient requires care of the provider. Ann Fam Med. 2014;12:573–576. doi:10.1370/afm.1713.

**Response:** We thank the reviewer for this important observation. We have revised this section to improve clarity, strengthen the transition, and more clearly distinguish between work-related perceptions and organisational outcomes. Specifically, we now explicitly clarify that these findings refer to participant-reported perceptions from Braining team members, rather than formally measured outcomes. The text has also been restructured to separate staff-related experiences (e.g., sense of purpose and enjoyment at work) from organisational-level observations (e.g., collaboration between the two units), thereby improving coherence. In addition, we have refined the wording to avoid implying measurement of job satisfaction or workload, and we have chosen to remove “stress”. Finally, we have verified and updated the reference to the Quadruple Aim framework (Bodenheimer & Sinsky, 2014).

Lines 591-607:

Moreover, Braining appeared to positively influence the participants’ perceived sense of purpose and enjoyment at work, raising the question of whether the innovation may also affect job satisfaction, work engagement, and perceived value among HCWs. These observations reflect participant-reported perceptions rather than formally measured outcomes. Such outcomes are particularly relevant given evidence that higher staff engagement is associated with reduced turnover and improved workforce productivity in healthcare settings (51).

An additional and unanticipated outcome, as reported by participants, was enhanced collaboration between the two units. According to participants, Braining strengthened peer-to-peer relationships and improved referral processes, suggesting that innovation may confer organisational benefits beyond patient-level effects. Such improvements in collaboration and coordination are consistent with implementation literature emphasising the role of organisational context and interprofessional collaboration in successful implementation (35). Taken together, these participant-reported findings highlight that HCWs may experience distinct, often overlooked outcomes, such as satisfaction and work experience with delivering an innovation. As emphasised within the Quadruple Aim framework, improving the work-life and well-being of HCWs is a critical consideration, as their experiences may influence the sustainability of innovations in real-world practice (52).
